# Epidemiologic characteristics of leprosy in Mainland China, 2004–2020

**DOI:** 10.3389/fpubh.2025.1666605

**Published:** 2025-09-24

**Authors:** Yuanchao Yang, Jiaqi Zeng, Jingyi Li

**Affiliations:** ^1^Department of Dermatology, Huizhou First Hospital, Huizhou, China; ^2^Department of Dermatology, Zhuhai People's Hospital (Zhuhai Clinical Medical College of Jinan University), Zhuhai, China

**Keywords:** China, disease burden, epidemiology, leprosy, *Mycobacterium leprae*

## Abstract

**Background:**

Leprosy is a chronic infectious disease caused by *Mycobacterium leprae*. This study aimed to elucidate the epidemiological characteristics of this condition in China from 2004 to 2020.

**Methods:**

New leprosy case and incidence data covering all 31 provinces in Mainland China from 2004 to 2020 were collected from the Data Center of China’s Public Health Science. The temporal, regional, and age-specific distributions of leprosy were analyzed through epidemiological methods.

**Results:**

From 2004 to 2020, a total of 5,478 leprosy cases were reported in China, with an average annual incidence rate of 0.0239 per 100,000 population, which indicates a low and stable endemicity. Geographically, the highest disease burden was observed in Yunnan, Guangdong, Sichuan, Guizhou, and Guangxi provinces. Regarding age distribution, the majority of cases were observed among individuals aged 30–49 years (2,171 cases, 39.63%), and the highest incidence rate was found in the 60–69-year age group.

**Conclusion:**

Leprosy incidence remained stably low nationwide and displayed significant regional variation. New cases clustered predominantly in Southwestern and Central–South China (Yunnan, Guangdong, Sichuan, Guizhou, and Guangxi). The disease burden showed distinct age patterns, with the highest case numbers detected in middle-aged adults and peak incidence rates in the older population. Targeted prevention strategies should prioritize these high-burden regions and peak-affected age groups.

## Introduction

Leprosy refers to a chronic infectious disease caused by *Mycobacterium leprae*, and it primarily affects the skin and peripheral nerves. Left untreated, this condition can lead to irreversible nerve damage and permanent disabilities (1). Currently, approximately 200,000 new leprosy cases are reported per year globally, and they are particularly prevalent in tropical and subtropical regions with poor sanitary conditions (2, 3). China once belonged to the high-burden leprosy countries; the epidemic has been alleviated after multidrug therapy (MDT), and China achieved the World Health Organization’s leprosy elimination goal, which is an incidence of less than 1/100,000 population in 1981 (4, 5). However, 200–400 new leprosy cases are still being reported annually over the past decade or more, and they remain a public health concern in several areas in China. This phenomenon may be closely related to the risk of imported cases, the emergence of MDT resistance, and delayed diagnosis in remote areas (6).

A series of policies and measures was formulated and implemented in China to control and eliminate leprosy: “National Plan for Leprosy Prevention and Control,” “National Regulations on the Administration of Leprosy,” “National Leprosy Prevention and Control Regulations for the Period 1985–2000,” “National Plan for the Prevention and Treatment of Leprosy (2001–2005),” and “Program for the Elimination of Leprosy in China (2011–2020)” (5). Jiang et al. reported leprosy epidemiological characteristics in China from 2004 to 2016 (6). However, policy and public health interventions can lead to shifts in epidemiological patterns. Therefore, strategies for the prevention and control strategy of leprosy to analyze the epidemiological characteristics of leprosy in China from 2004 to 2020 have great public health significance.

## Materials and methods

### Data source

New cases and incidence data on leprosy were obtained from the Data Center of China’s Public Health Science^1^; they encompassed all 31 provincial-level administrative divisions (including provinces, autonomous regions, and municipalities) in Mainland China from 2004 to 2020. The Data Center of China Public Health Science is a national scientific data-sharing project initiated by the Ministry of Science and Technology of the People’s Republic of China in 2004. The database contains all reported leprosy cases since the direct network reporting of infectious diseases in 2004 and provides pre-calculated data including case counts and incidence rate (per 100,000). The cases met World Health Organization’s diagnostic criteria for leprosy and confirmed through laboratory testing or clinical examination by designated dermatologists. The dataset contains aggregated epidemiological information without patient detail. No ethical permission is required for publicly downloaded data.

### Statistical analyses

All statistical analyses were performed using Microsoft Excel 2019 (Microsoft Corp., USA), and data visualization was conducted using Origin 2021 (OriginLab Corp., USA) and ArcGIS Pro 2023 (Esri Inc., USA).

## Results

### Epidemic situation

**Figure 1:**
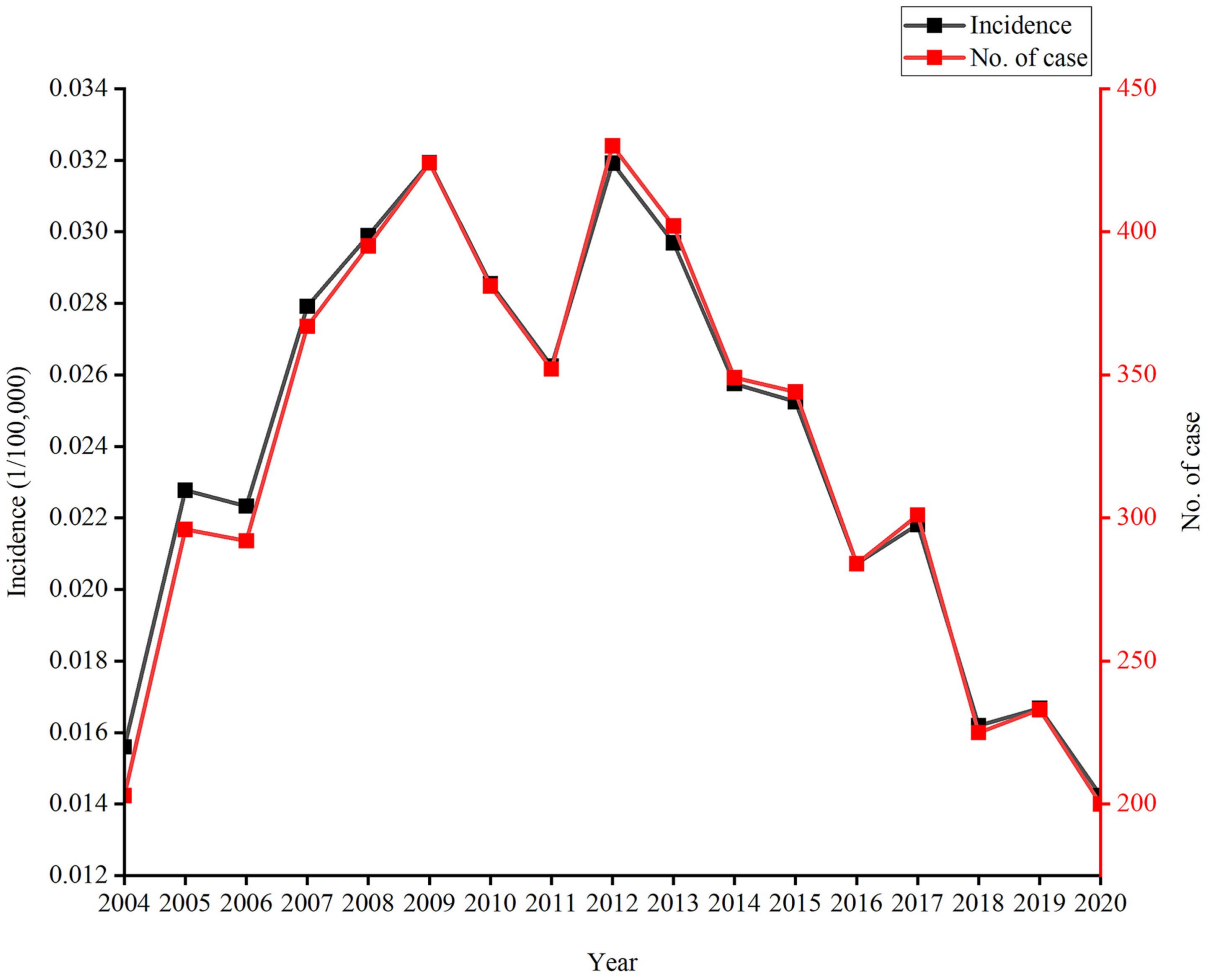
During 2004–2020, incidence rates and new leprosy cases exhibited an increase-then-decrease pattern in China.

Almost 200–400 new cases of leprosy are reported annually, and 5,478 leprosy patients are recorded in China, with an average annual incidence of 0.0239/100,000 during 2004–2020. The overall trend in the incidence of leprosy was stable and indicated a low incidence from 2004 to 2020. However, the incidence rate increased slightly from 2004 to 2013 and then decreased to some extent. The leprosy incidence was from 0.0156 per 100,000 people in 2004 to 0.0142 per 100,000 persons in 2020 (Table 1 and Figure 1).

**Table 1:** Epidemic situation of Leprosy in China from 2004 to 2020.

Year	No. of case	Incidence (per 100,000 population)	Growth rate
2004	203	0.0156	–
2005	296	0.0228	0.4597
2006	292	0.0223	−0.0193
2007	367	0.0279	0.2502
2008	395	0.0299	0.0707
2009	424	0.0319	0.0680
2010	381	0.0285	−0.1059
2011	352	0.0263	−0.0804
2012	430	0.0319	0.2158
2013	402	0.0297	−0.0697
2014	349	0.0258	−0.1326
2015	344	0.0252	−0.0196
2016	284	0.0207	−0.1794
2017	301	0.0218	0.0529
2018	225	0.0162	−0.2575
2019	233	0.0167	0.0300
2020	200	0.0142	−0.1461
Total	5478	0.0239^a^	–

### Region distribution characteristics

**Figure 2:**
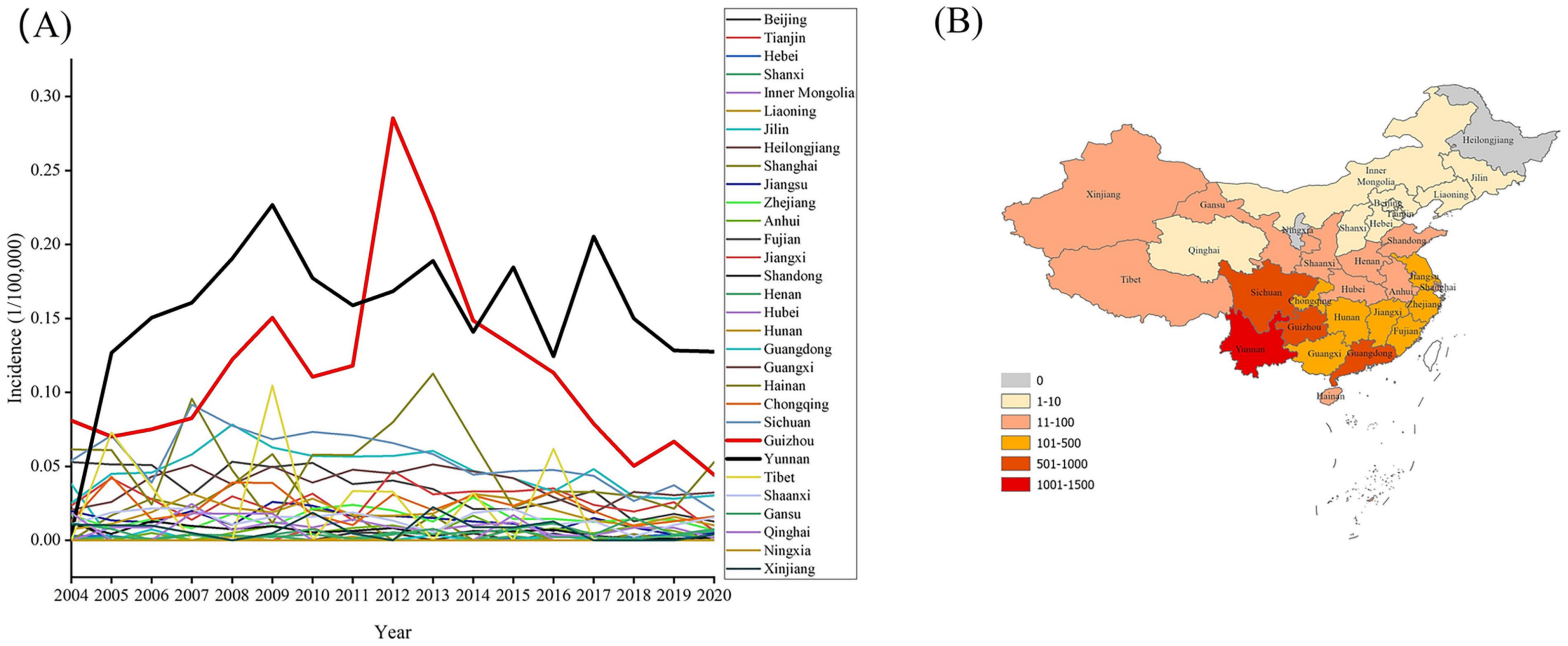
During 2004–2020, incidence rates **(A)** and distribution of new leprosy cases **(B)** varied widely by district in China.

Across China, new case and incidence rates of leprosy varied widely by district. In 2004–2020, the top five provinces or municipalities with the highest number of new cases of leprosy included Yunnan, Guangdong, Sichuan, Guizhou, and Guangxi. These regions accounted for 69.37% (3800 cases) of the total number of cases. In terms of average incidence, the top five districts were Yunnan (0.1538/100,000), Guizhou (0.1147/100,000), Sichuan (0.0551/100,000), Hainan (0.0511/100,000), and Guangdong (0.0473/100,000). Most regions, which included Beijing, Tianjin, Hebei, Shanxi, Shaanxi, Heilongjiang, Jilin, Liaoning, Inner Mongolia, Qinghai, Gansu, Ningxia, and Xinjiang, had fewer than 10 new cases annually during the study period. Specifically, no leprosy case was reported in Heilongjiang and Ningxia during the study period. In 11 out of 31 provincial administrative regions in Mainland China, the total number of leprosy cases was more than 100 (Figure 2).

### Age distribution characteristics

**Figure 3:**
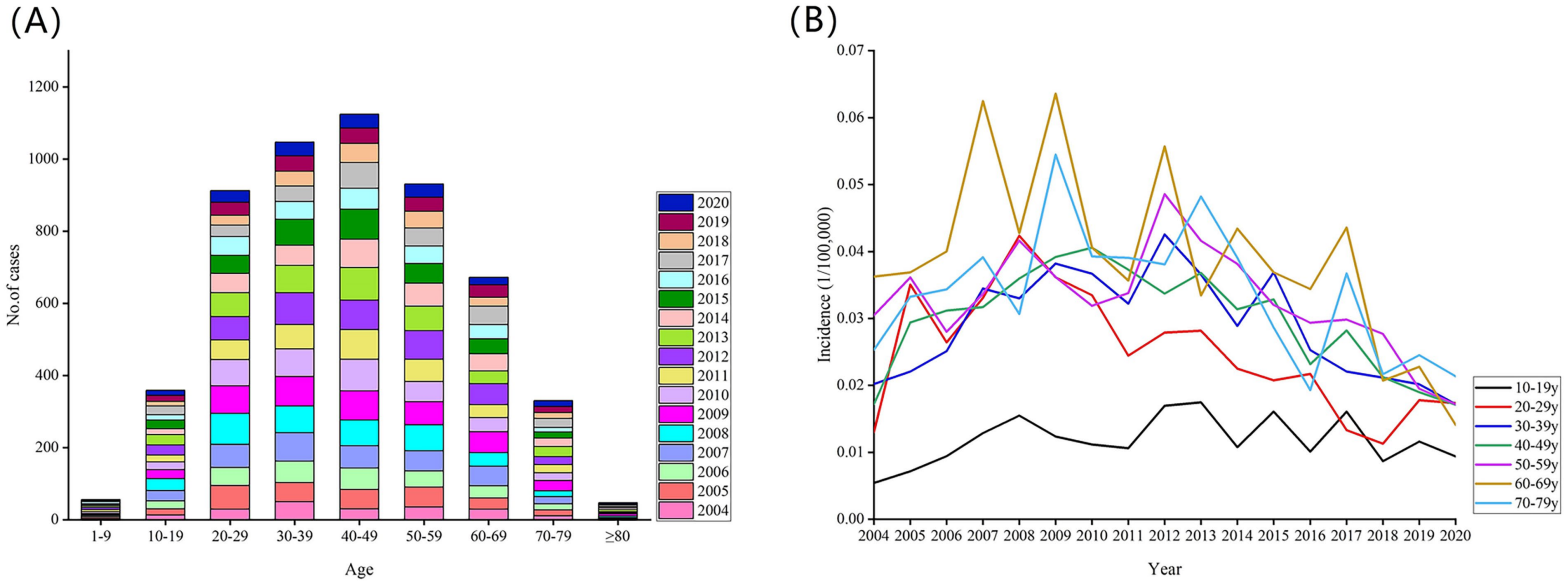
During 2004–2020, age distribution of new leprosy cases **(A)** and incidence rates **(B)**.

During 2004–2020, most of the cases were aged 30–49 years (2171 cases, 39.63%), 56 (1.02%) were under 10 years, 359 (6.55%) were 10–19 years, 912 (16.65%) were 20–29 years, 1047 (19.11%) were 30–39 years, 1124 (20.52%) were 40–49 years, 931(17.00%) were 50–59 years, and 1049 (19.15%) were aged 60 years or older. In terms of incidence, people in the 60–69-year age group had a distinct peak incidence (0.0374/100,000), followed by those in the 70–79- (0.0331/100,000) and 50–59-year (0.0323/100,000) age groups (Figure 3).

## Discussion

To date, leprosy is never fully eradicated, and approximately 200,000 new cases are reported per year globally (3). Although, the leprosy incidence rate in China has been consistently low, and leprosy was eliminated in China according to the World Health Organization (elimination standard defined as incidence less than 1/100,000) (1, 5), 200–400 new cases are still reported annually, which may pose a challenge to its control and prevention (5). In 2004, China included leprosy prevention and treatment in its national public health program, with sustained government funding at all levels. Currently, leprosy diagnosis and treatment are free nationwide (5). In 2011, China issued the Program for the Elimination of Leprosy in China (2011–2020), which focused on controlling leprosy incidence and its harms through direct public health investments (7). The epidemiological characteristics of leprosy cases in China from 2004 to 2016 have been reported previously; the incidence remained relatively stable over time, with the total incidence reaching 0.02815/100,000 (6). As a result of policy and public health interventions, the epidemiological characteristics of leprosy may be changed. In this study, the longitudinal surveillance data of leprosy in China from 2004 to 2020 was analyzed to identify the epidemiological characteristics for effective prevention and management planning for leprosy.

In our study, the upward trend of incidence rates and new cases observed from 2004 onward can be attributed to the following reasons: First, the timeliness of reporting has been improved since the direct network reporting of infectious diseases; second, the advancements in diagnostic technology and laboratory testing have resulted in an increased case detection. However, a downward trend emerged after 2013, which can be due to the implementation of “Program for the Elimination of Leprosy in China (2011–2020),” which strengthened leprosy prevention and control measures across the country. Since the implementation of the program, 98% of cities in China have achieved the goal of controlling the leprosy incidence rate below 1/100,000 (5). A total of 5,478 new leprosy cases were recorded during 2004–2020, with the incidence rate remaining relatively stable (0.0142–0.0319/100,000). In addition, a low incidence (average annual incidence 0.0239/100,000) was observed, with the value being lower than the global average (2.29/100,000) in 2019 (2), such as those of Pakistan (0.11/100,000) in 2022 (8) and Brazil’s overall new case detection rate (10.3/100,000) in 2017 (9). This achievement can be attributed to the vigorous promotion of MDT, relevant public health policies, and investment (5, 10).

In the last century, more than 500,000 leprosy patients were recorded in China, and they were mainly concentrated in Yunnan, Guizhou, Sichuan, and Jiangsu (11). Leprosy showed a heterogeneous distribution in China, with Yunnan, Guangdong, Sichuan, Guizhou, and Guangxi provinces having with the most leprosy cases (concentrated in southwest and central-southern region), consistent with previous work (6). The clustering areas of leprosy were mostly distributed in the border areas of Yunnan and Guizhou, and the secondary clustered district was found in the western region (12). This finding may be related to environmental factors, economic development, population migration, and border regions. Soil properties, humidity, vegetation, and hydrothermal conditions may serve as potential vectors for leprosy transmission (13). Previous studies have shown that leprosy cases in Southwestern China were most prevalent in poor regions with difficult terrain, which indicates an association between leprosy and poor living environments and transportation systems and weak economies (14). China is geographically adjacent to India, Nepal, and Myanmar, which are leprosy-focus 23 countries; therefore, overseas imported cases may play a certain role in the prevalence of leprosy. Population migration is an important factor in the transmission of leprosy, which primarily occurs in the low-resource setting rural and urban areas (15). Migration can cause environmental changes, which influences disease transmission and the related risk, with approximately 1/100,000 cases occurring among internal migrants each year and new cases appearing in international migration (10). From 2011 to 2018, 11.5% newly detected leprosy cases in China were detected in individuals who had migrated from traditionally leprosy endemic to relatively developed cities, such as Beijing, Shanghai, and Guangzhou (10). From 2011 to 2019, 85.16% of leprosy cases among the floating population in Zhejiang came from the southwest region (Guizhou, Yunnan, and Sichuan) (16). During 2001–2021, 22.8% of leprosy patients in Guangdong migrated from Hunan, Jiangxi, and the southwest region (15). In addition, 15.15% of leprosy patients in Jiangsu were from other regions, predominantly the high-endemic provinces of Guizhou and Sichuan (11). Therefore, for medium- and low-risk regions, current management protocols must be maintained while implementing strict measures to prevent the importation of leprosy cases from high-endemic areas. Moreover, in medically underserved areas (such as Yunnan and Guizhou), delay in diagnosis results in concealed infection sources and undetected transmission.

Otherwise, middle-aged adults (aged 30–49 years) exhibited most leprosy cases (2171 cases, 39.63%), and the older adults (aged 60–69 years) had a high leprosy incidence rate, which highlights the need for targeted prevention and control measures for both population groups. A similar situation was reported from Brazil: 19,582 (24.1%) leprosy cases occurred in individuals aged 60 years and above, 30,593 (37.7%) in the 40–59 age group, 25,896 (31.9%) in those aged 15–39 years, and 5,134 (6.3%) in children under 15 years old from 2016 to 2018 (17). This finding was possibly due to the long incubation period of leprosy and the frequent social activities of middle-aged adults increasing their exposure risk (6); in addition, the older adults had poor knowledge about leprosy, declined immune system, long latency period, and were underdiagnosed in their youth (18). In our study, middle-aged adults accounted for the majority of leprosy cases in absolute numbers, and the older population exhibited a high incidence rate. This pattern may be attributed to China’s demographic structure, where the absolute number of middle-aged individuals is substantially large, which led to a high case count in this group (19). Hence, focused interventions among middle-age and older adults must be strengthened. A total of 82.2% leprosy cases were identified from dermatological consultation, and 17.8% were identified from contact, epidemic point investigation, self-reporting, mutual reporting, or census (15). Dermatology clinics serve as the primary setting for leprosy detection. Dermatologists must be aware that leprosy may persist in low-endemic settings. Therefore, regular training for dermatologists is essential to ensure early case detection.

In China, 200–400 new cases of leprosy are reported annually, and the incidence rate remains stable and low. The epidemics of leprosy varies considerably among regions, and new cases are mainly distributed in the southwest and midsouth regions (Yunnan, Guangdong, Sichuan, Guizhou, and Guangxi). Most cases occurred in adults aged 30–49 years, and the highest incidence rates were observed in those aged 60–69 years. These epidemiological findings may inform targeted prevention strategies, which should prioritize high-burden regions (southwest/mid-south) and age groups (30–69 years), combined with innovative and effective methods to enhance early diagnosis and reduce transmission. Aligning interventions with these epidemiological patterns will accelerate progress toward elimination.

Although we have revealed crucial results, our study still encountered limitations. First, the data were fairly limited, and leprosy incidence was not disaggregated by gender, occupation, or degree of disability, which can provide deeper insights into risk factors. Second, the study period (2004–2020) did not cover the COVID-19 pandemic years (2020–2022) as our analysis relied on publicly available data from the Data Center of China’s Public Health Science (updated through 2020). Thus, we were unable to assess the potential effect of the COVID-19 pandemic on leprosy surveillance and case reporting. Although we failed to obtain the above details from existing databases within a short time frame, we will still strive to collect related data and analyze other factors relative to leprosy incidence to optimize prevention and control policies in the future.

## Data Availability

The original contributions presented in the study are included in the article/supplementary material, further inquiries can be directed to the corresponding author.

## Footnotes

^1^ https://www.phsciencedata.cn/Share/index.jsp
